# Irritability and Risk-Taking Behaviour on the Cambridge Gambling Task (CGT) in Adolescents With a Family History of Depression

**DOI:** 10.1192/bjo.2024.250

**Published:** 2024-08-01

**Authors:** Ramya Srinivasan, Glyn Lewis, Jon Roiser, Frances Rice

**Affiliations:** 1 UCL, London, United Kingdom; 2 Cardiff University, Cardiff, United Kingdom

## Abstract

**Aims:**

Irritability is a common symptom in children and adolescents, often resulting in referral to mental health services and is associated with depression. Depression in adolescents and adults at familial risk of, and with depression, is associated with reduced risk-taking on the Cambridge Gambling Task (CGT) particularly when the chance of winning is high. However, little is known about risk-taking in irritability. This study tests the hypothesis that increased irritability is longitudinally associated with later risk-taking behaviour on the CGT; specifically, that increasing irritability is associated with lower risk-taking when the chance of a favourable outcome is high.

**Methods:**

We conducted a longitudinal study of the biological offspring of parents of children with depression (n = 337). Irritability, the exposure, was measured at wave one using the Child and Adolescent Psychiatric Assessment (CAPA). The primary outcome was risk-taking to obtain reward at varying probability ratios (6:4, 7:3, 8:2 and 9:1) measured by the Cambridge Gambling Task (CGT) at waves two and three. We investigated the longitudinal association between irritability at wave one and average risk-taking at each ratio across waves two and three using multi-level models. The extent to which risk-taking according to probability ratio varied with irritability was tested with interaction terms. We ran univariable models and then multivariable models.

**Results:**

In univariable (n = 207; Coef. 0.006, 95%CI −0.011–0.023, p = 0.470), and fully adjusted (Coef. 0.011, 95%CI −0.007–0.029, p = 0.213) models there was no evidence of a main association between irritability and risk-taking on the CGT. There was evidence of an interaction between irritability and risk-taking ratio (p = 0.019). In fully adjusted models including the interaction, a one-point increase in irritability was associated with relatively higher risk-taking at the less favourable ratios (6:4 – 0.018 (95%CI −0.002–0.037) and 7:3 – 0.015 (95%CI −0.005–0.035)) relative to the more favourable ratios (9:1 – 0.001 (95%CI −0.019–0.021) and 8:1 – 0.011 (95%CI −0.008-0.031)).

**Conclusion:**

We found no evidence of relationship between irritability and subsequent risk-taking on the CGT overall. However, there was some evidence that those with higher irritability were relatively more risk-taking when less likely to win compared with when a favourable outcome was more likely. These findings warrant further investigation of the association between prior irritability and later depression in a larger community cohort. If prior irritability and depression are both associated with risk-taking, this strengthens the case for focussing on risk-taking as a potential target for preventive intervention.

